# Volumetric Assessment of Apical Periodontitis Using Cone-Beam Computed Tomography—A Systematic Review

**DOI:** 10.3390/ijerph20042940

**Published:** 2023-02-08

**Authors:** Ewa Mackiewicz, Tobias Bonsmann, Kinga Kaczor-Wiankowska, Alicja Nowicka

**Affiliations:** 1 Student Scientific Circle at the Department of Conservative Dentistry and Endodontics, Pomeranian Medical University in Szczecin, Powstancow Wielkopolskich 72, 70-111 Szczecin, Poland; 2 Department of Conservative Dentistry and Endodontics, Pomeranian Medical University in Szczecin, Powstancow Wielkopolskich 72, 70-111 Szczecin, Poland

**Keywords:** apical periodontitis, cone-beam computed tomography, systematic review, volumetric studies

## Abstract

This systematic review aimed to investigate the scientific literature on volumetric studies concerning the diagnosis and treatment of apical periodontitis using CBCT. A systematic review protocol was written following the preferred reporting items for the systematic reviews and meta-analyses (PRISMA) checklist. Four electronic databases were searched for relevant publications in English, which were published up to 21 January 2023. The inclusion criteria and corresponding search keys were applied. The risk of bias was assessed using the Joanna Briggs Institute Meta-Analysis of Statistic Assessment and Review Instrument. The search strategy identified 202 studies, with 123 studies excluded during the title and abstract screening and 47 studies left for full text screening. A total of 17 studies met the inclusion criteria. The lesion volume was measured and classified according to different indices which compared the effectiveness of their diagnostics. Moreover, the volume of AP lesions increased with the thickness of the maxillary sinus mucosa in primary and secondary infections and decreased due to endodontic treatment. Volumetric measurements using CBCT are useful in the correct definition of periapical tissue pathosis using a CBCT periapical volume index and assessment of the dynamics of the treatment of apical lesions.

## 1. Introduction

Apical periodontitis (AP) is the inflammation and destruction of periradicular tissues which occurs as a sequence of various insults to the dental pulp, including infection, physical, and iatrogenic trauma, and damaging effects of root canal-filling materials following endodontic treatment [1]. Periapical periodontitis leads to an osteolytic process, which becomes visible after a period of time on two- (2D) and three-dimensional (3D) radiographic documentation as a radiolucent field compared to the surrounding healthy tissue structures [2].

The radiological detection of the changes in the apical tissues using cone-beam computed tomography (CBCT) imaging helps endodontists determine the lesions and pathological changes in the patient [3]. Moreover, CBCT is more accurate and reliable compared with 2D radiography [4,5,6,7,8,9]. Volumetric dimensions can also be visualized, which is not possible in 2D images [7]. The use of CBCT in endodontics is increasing rapidly worldwide and is reflected in position statements published by several specialist societies (European Society of Endodontology 2014, American Association of Endodontists/American Academy of Oral & Maxillofacial Radiology CBCT position statement 2015) [6].

One of the main values for a successful outcome after a performed endodontic treatment is the recession and/or complete healing of intra-bony lesions caused by AP [7]. Different software programs, such as Amira [10,11,12,13,14,15,16], Mimics [17,18,19], and 3D Doctor [20,21], are designed for volumetric interpretation on CBCT-based data. They allow the practitioner to evaluate the outcome of treatment through assessments of volumetric changes which are measured and compared over given periods of time.

The volume of the lesion, together with the tooth position, is far more important than the shape of the lesion, as it influences the identification of the lesions. Overall, 33% of the lesions with volumes 6.7 mm^3^–41.3 mm^3^ were not identified on the periapical radiograph but were with the usage of CBCT [18]. Furthermore, the volume of the lesions helps in differential diagnosis of the pathosis with endodontic origin due to their known characteristic shape and sphericity of the lesions [19]. Moreover, volumetric measurements are improving the knowledge about expansion of the lesions, thus enabling clinicians to perform detailed periapical surgery with better vision [19]. Therefore, volumetric measurements are more indicated concerning diagnostic purposes, assessment of the healing, and the prognosis of the treatment [19].

Many systematic reviews about AP concerning CBCT have been reported [4,5,6,7,8,9]. Most of these compared 2D and 3D views and reported that CBCT has twice as good detection of apical changes than a periapical radiograph. Nowadays, due to the recent and ongoing high interest in the topic of volumetric measurements, the necessity of publishing a detailed review dealing with issue of determination of periapical lesion volume is clearly given. Studies involving volumetric measurements have never been analyzed so far, and to the best of our knowledge, this is the first systematic review on the volume of apical lesions. This systematic review aimed to investigate volumetric studies of periapical lesions that used CBCT and underline the importance of the volumetric measurements in the diagnosis and treatment of apical periodontitis.

## 2. Materials and Methods

**Figure 1:**
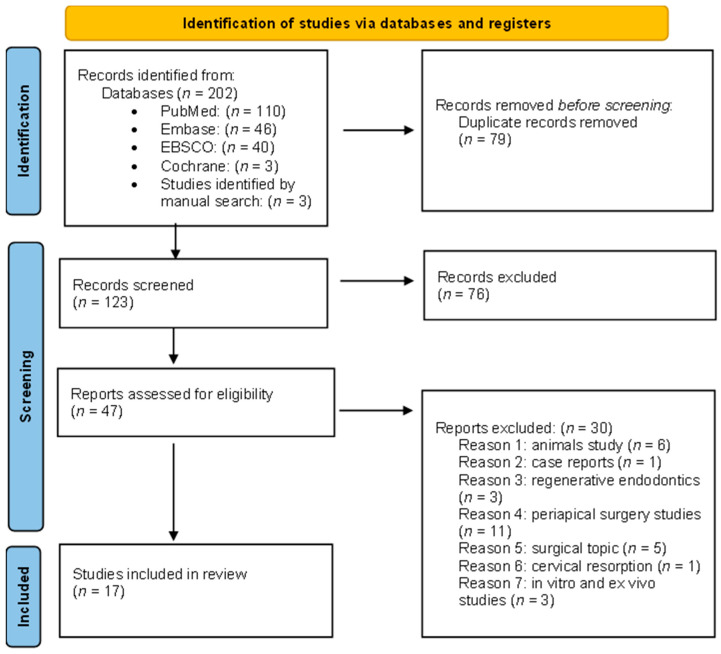
Preferred Reporting Items for Systematic Reviews and Meta- Analyses flowchart of study selection.

This study complies with the Preferred Reporting Items for Systematic Reviews and Meta-analysis Statement (PRISMA) [22] (Figure 1). The protocol of this systematic review was registered in the PROSPERO database (CRD42023392865).

### 2.1. Study Selection Criteria

The inclusion criteria were studies which (1) were published from the inception of the databases to 21 January 2023; (2) in vivo (humans); (3) included patients with AP which was treated, with the lesions documented using CBCT concerning volumetric dimensions before treatment and after a follow-up time; (4) included the use of a program for the exact determination of the 3D measurements of apical lesions through CBCT; and (5) assessed the effect of volumetric measurement on the judgement of the clinician in cases of endodontically treated teeth with an apical lesion.

### 2.2. Exclusion Criteria

The exclusion criteria were studies which (1) included animals; (2) were in vitro or ex vivo; (3) wherein periapical volumes have been determined without a program for volumetric calculations in three dimensions; (4) did not provide exact data about the volumetric assessment of measured lesions; (5) referred to a dental surgery branch; (6) were case reports; (7) referred to a cervical resorption and (8) to regenerative endodontics topic.

### 2.3. Search Strategies

The search was conducted independently by two reviewers (E.M and T.B). The following electronic databases were searched from their inception to 21 January 2023: PubMed accessed on 21 January 2023 (https://www.ncbi.nlm.nih.gov/pubmed), EBSCO Dentistry and Oral Sciences Source accessed on 21 January 2023 (https://www.ebsco.com/products/research-databases/dentistry-oral-sciences-source), EMBASE accessed on 21 January 2023 (https://www.embase.com/), and Cochrane Library accessed on 21 January 2023 (https://www.cochranelibrary.com/) (Table 1). Supplemental research was performed by screening the reference section of the relevant studies which were eligible for inclusion in the present systematic review. Articles that resulted from the search strategy were first screened based on the relevance of the title and abstract and reviewed and rejected if one of the exclusion criteria was met. In the second screening, full-text articles were then reviewed to ensure that they met the inclusion criteria. In the case of disagreement, a consensus between the reviewers was obtained through discussion or involving a third reviewer (A.N).

### 2.4. Quality Assessment

The methodology of the selected studies was evaluated using the Joanna Briggs Institute Meta-Analysis of Statistic Assessment and Review Instrument [23]. The risk of bias was determined by answering the following nine questions: (1) Was the study based on a random or pseudorandom sample? (2) Were the criteria for inclusion in the sample clearly defined? (3) Were confounding factors identified and strategies to deal with them stated? (4) Were outcomes assessed using objective criteria? (5) If comparisons were made, were the groups sufficiently described? (6) Was follow-up conducted over a sufficient time period? (7) Were the outcomes of people who withdrew described and included in the analysis? (8) Were the outcomes measured in a reliable way? and (9) was an appropriate statistical analysis used? Afterwards, the “yes’’ answers were summarized, and studies were classified as: “H’’—high (0–3), “M’’—moderate (4–6), and “L’’—low (7–9).

## 3. Results

### 3.1. Study Selection

The searches in PubMed, EBSCO Dentistry and Oral Sciences Source, EMBASE, and Cochrane Library yielded, 110, 46, 40, and 3 articles, respectively, as well as 3 studies identified by hand search, for a total of 202 records. Subsequently, 79 duplicates were removed, resulting in a total of 123 articles. After the first screening (title and abstract), 76 records were excluded. Afterwards, the remaining 47 reports were subjected to full-text review for eligibility assessment. Moreover, 30 additional articles were excluded because they did not meet the inclusion criteria: six were animal studies [24,25,26,27,28,29], one was a case report [30], three dealt with regenerative endodontics [31,32,33], 16 were based on a surgical topic [2,34,35,36,37,38,39,40,41,42,43,44,45,46,47,48], one dealt with cervical resorption [49], and three were in vitro studies [50,51,52]. Thus, the final inclusion for this qualitative review comprised 17 articles.

### 3.2. Study Characteristics

The 17 reports selected were published between 2013–2021. All studies included patients with apical periodontitis which were documented via CBCT. They reported the measured volume of periapical lesions: two studies combined the volumetric measurements of periapical lesions, as well as those of the maxillary sinus mucosa [3,20], whereas eleven studies used CBCT to measure the apical lesion volume solely to distinguish healing outcomes and/or compare it with other dental imaging techniques [10,11,12,15,16,17,21,53,54,55,56], two studies used the measured volume to evaluate the accuracy of periapical indices [13,18], and one study compared CBCTPAVI index to the sphericity of the lesions [19]. The key point investigated was the volumetric determination of apical lesions and their alteration in size and dimension after successful endodontic treatment in different follow-up time spans, alongside the comparison of accuracy between 3D and 2D radiographic measurements in dentistry. The target conditions and purpose of the volumetric determination of lesions measured in each of the included studies are presented in Table 2. The examined groups in the studies have been mostly females with ages of more than 18 and 79 years old at most. All of the studies’ target condition was periapical lesion mostly in molars. The selected studies had different groups of treated patients and their teeth, observational times, used programs, and outcome measures. Due to the substantial heterogeneity noted in the included preclinical studies, a quantitative data synthesis for a meta-analysis cannot be conducted.

### 3.3. CBCT Parameters

In the majority of publications, the CBCT apparatus was used in mostly different ways; however, in four studies, I-CAT was used [12,13,53,54]. Voltage varied from 60–120 kV, and the current ranged from 2 to 15 mA. The voxel size in most publications accounted for 0.2 mm^3^. The thickness of the layer was not mentioned in most studies; however, they accounted for 0.2 mm. Most common segmentation methods were manual [14,16,20,21] and automatic [12,13,17,53]. Semiautomatic segmentation was done in two publications [10,11]. However, there was only given information concerning the segmentation time in one of the studies [54]. Different programs have been used for CBCT reading and volume calculations. The Amira software has been used in seven studies [10,11,12,13,14,15,16]. Mimics has been used in three publications [17,18,19], and 3D Doctor and ITK SNAP software have been used in two publications each, respectively [20,21,56,57], and single studies used: Planmeca Viewer [3], Nemotec [54], OsiriX [54] and Implant Viewer [54]. Data on the CBCT parameters in the analysed studies are presented in Table 3.

### 3.4. Results of Individual Studies

**Figure 2:**
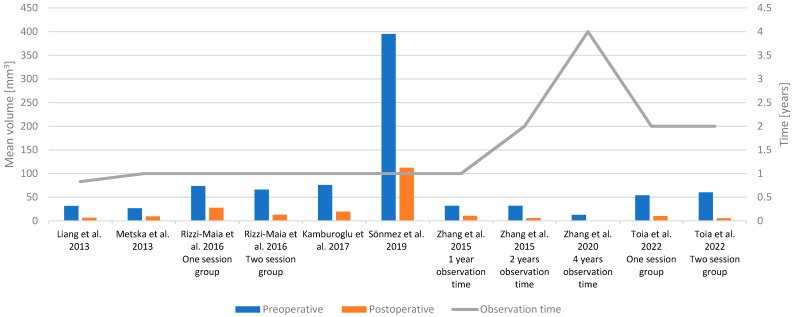
Postoperative decrease of the volume of apical changes in different observation times [10,11,12,20,21,16,14,56].

Treatment success was compared with the volume of periapical lesions in most of the included publications. Table 4 summarizes the results of the included studies. Decreased volume after treatment suggests the successive healing of the lesion. The observation times of research lasted from 6 months to 4 years. Secondary endodontic infections have been connected with the higher mucosal thickening of the maxillary sinus, as well as with the larger volume of periapical lesion compared to that of primary infections [3]. Furthermore, mucosal thickening was also reduced, together with the volume of the periapical lesion after successive endodontic treatment [20]. Two studies analysed whether single or two-visit treatment is more sufficient in treatment of necrotic pulp with visible periapical lesions and posttreatment apical periodontitis, respectively [12,56]. In the majority of the studies, the observation was 1 year [11,12,15,20]. After one year of observation time, healing progressed but was not completed in both single and two visits of root canal treatment [12]. However, in one study, it was prolonged to four years [14]. After 4 years of observation, 75.9% of the lesions disappeared completely [14]. In Figure 2, the preoperative and postoperative volume in a particular time period in chosen publications was illustrated.

Other studies analysed endotoxins levels [53], automated volumetric measurements [54], size and pattern of bone loss in patients with acute and chronic apical abscess [17], and diagnostic potential of high-resolution ultrasound with CBCT in assessing granulomas and radicular cysts [21].

The volume of the lesions with particular periapical volume index (PAVI), CBCTPAVI scores was measured, and the total mean volume was calculated [13,18]. Because of the large variations within the CBCTPAVI score before, the index was modified using the partition classification analysis, which gave the particular scores presented in Table 4. Furthermore, CBCTPAVI index with usage of volumetric measurements was used to determine the sphericity of the periapical lesions [19].

### 3.5. Quality Assessment

According to the Joanna Briggs Institute Meta-Analysis Statistics Assessment and Review Instrument, the bias within the studies was low for randomized studies [10,12,57] and moderate for the rest of the publications (Table 5). 

## 4. Discussion

This systematic review focused on evaluating existing scientific literature that dealt with volumetric determination and measurement of dental periapical tissue lesions caused by varied factors that differed in origin and expansion. They were achieved with the help of CBCT, which can be extremely useful in future diagnostics and treatment success prediction and observation in endodontics. Volumetric measurements are simple to achieve and they can be extremely useful. The operator has to manually determine the periapical lesion on the CBCT view of each layer. Often this procedure takes a lot of time, due to difficulties in assessing whether the lesion is already present or not. The room where the dentist is marking the lesion has to be properly dark to have a possibility of correctly grading the apical change. The manual contouring of the lesions is very laborious and often can be controversial, however in the future it is going to be exchanged by the automatic one which will be unified and not dependent on the dentist eye, only because it is believed that artificial intelligence in radiography and CBCT can clarify the border of the lesion and 3D assessment of the lesion [14]. Moreover, automated segmentation using a region growing algorithm took less time in measuring volume of the lesion and was more precise comparing to the manual one [54]. However, Aoki et al. [54] analysed the time of the segmentation. Manual segmentation was completed within 120 s in contrast to 50 s in automated one (Table 3). A very useful tool can be a vector-based volume rendering software, which determines the volumetric changes of periapical lesions after endodontic treatment [20].

For many different operator systems in radiographic diagnostics with the help of CBCT, as well as in data progression software and operating systems, distinct ways and outcomes of volumetric evaluations are stated in literature. Programs enabling the operator to determine the Volume like OsiriX, Mimics, Amira, 3D Doctor, etc., do not only aid in determining correct initial lesion size [13,18] and origin [3,53], but furthermore allow the comparison of different treatment approaches in accordance to heal the apical periodontitis. However, CBCT examination, which is necessary for the volume calculations, generates quite high ionization, but the effective dose of ionization can be nearly as low as a panoramic dental X-ray and considerably less than a medical CT scan [54]. It is essential that patient radiation exposure is kept as low as reasonably practicable and that evidence-based selection criteria for CBCT use are developed [55]. As low as reasonably achievable (ALARA) principles should be maintained during all dental diagnostic imaging [56]. Although the radiation dose may be further reduced by decreasing the size of the field of view, increasing the voxel size and/or reducing the number of projection images taken as the X-ray source rotates around the patient [57]. In publications where the CBCT was compared to the USG, the depth, surface area, and the volume of the lesion were significantly lowered compared to CBCT. This difference can appear due to the buccal cortical bone measurements [21]. It was stated that the AP volume measurements by CBCT strictly rely on the criteria defined by radiolucency [12].

CBCT has twice the probability to detect periapical lesions. A two-dimensional radiograph, in contrast, has lower sensitivity in the diagnostics [9,58,59,60]. The studies evaluated radiological indicators that help the dentist in proper diagnosis [13,18,61,62], and one of them is the Periapical Index Score (PAI), which is often used in everyday practice [61]. PAI is a scoring system for evaluating apical periodontitis via radiographs [62]. It uses a scale from 1 (healthy periodontal tissue) to 5 (severe periodontitis). Unfortunately, it does not take into consideration the volumetric characteristics of the periapical lesions, thus it is not the most reliable tool used in diagnostics [61]. Additionally, lesion volume is the determining factor of choice regarding PAI score, however information about reduced lesion from PAI score does not mean a simultaneous reduction of lesion volume. It is influenced by the overlapping of additional tissues, density and thickness of the bone cortex, and the distance between lesion and cortical bone. It is suggested that the only tool which is properly measuring the reduction of the periapical lesion is CBCT [13]. The CBCTPAI scores 3, 4, and 5 showed the high variances in the volumes of lesions, which is why the new CBCTPAVI (cone-beam computed tomographic periapical volume index) was announced [18]. It was suggested that due to the development of the volume-based CBCTPAI, which allows for very accurate volumetric determination, the operator is allowed to estimate the healing outcome far more precisely [18,62].

Moreover, a study on epidemiologic data [8] stated that the worldwide prevalence of AP slightly increased, and that patients older than 50 years old are more likely to develop AP, while women are less prone to develop AP. In the analysed studies, the authors assessed two methods of treating periapical lesions: single sessions treatments [10,14] and two sessions treatment (calcium hydroxide used as a dressing between the visits) [20,21]. Single sessions treatments have been used to observe if healing with or without ultrasonic activation is more effective, and it appeared that both activation and no activation of the irrigant contributed equally to the healing of periapical lesions [10], as well as to determining the healing after 4 years of observation (75,9% of completely healed lesions) [14]. The treatment observations of two sessions resulted in a reduction of the periapical lesions, but the research lasted for 1 year [20,21]. Moreover, in the one included study, the volumetric measurements have been used to determine whether one session or two sessions treatments are more effective [12]. Chemo-mechanical preparation was the same for both groups: ProTaper Universal System, irrigation with 5.25% NaOCl, and at the end EDTA placement for 3 min [12]. It appeared that one year time is not enough to show the complete healing of the periapical lesion, and in both, one session treatment and two session treatment similar lesions volume was observed, however more advanced reduction of the lesion appeared in the teeth which underwent the two-session treatment. Therefore, the estimated volume of periapical lesions can be a deciding factor as to whether the clinician should perform one visit or two visits treatment protocol. The bigger volume of the lesion suggests that the clinician should perform a two-visit root canal treatment. In addition, volumetric measurements also affect the treatment choice, due to the more accurate planning of the periapical surgery. The volume of the lesion can be used by the clinicians to obtain direct information about the spread and expansion characteristics of the lesions helping in performing more accurate access and vision, thus increasing the success rate of the procedure [19].

Due to the possibility of monitoring volumetric changes, we were able to prove that secondary endodontic infections showed a bigger lesion size than primary ones, and that the dimension of the cortical bone is in direct relation to the thickening of the maxillary sinus membrane [3]. The authors found a significant reduction in periapical lesion width, lesion height, surface area, and volume in maxillary molar teeth, along with adjacent sinus mucosal thickening 1 year after endodontic treatment [20]. The periapical lesion with adjacent mucosal thickening was treated in a two-session treatment with the calcium hydroxide dressing between visits, and root canals finally got obturated in single cone gutta percha technique [20]. In publications which analysed endotoxins levels [53], compared primary and secondary periapical lesions [3], automated volumetric measurements [54], radiologic indices [13,18], and dynamics of bone loss [17], the treatment was not performed. Authors evaluated the bone loss in acute (AAA) and chronic (CAA) apical abscesses. The median volume of AAA was 109 mm^3^ and for CAA it was 233 mm^3^. Furthermore, fenestration was present in 100% of CAA and in 47.8% of AAA cases [17]. In the study assessing the endotoxins levels, it was stated that the endotoxins and bacteria level in the root canal are directly proportional to the volume of the apical lesion [53].

The high sensitivity of CBCT regarding any changes, especially in volume, helps the operator to monitor the outcome after treatment at any point of recall time [10,11,12,14,16,17,20,21]. In periapical radiography, changes after a specific recall time may be seen but the possibility of false reflection of the true healing outcome, due to angulation or superimposition of anatomic landmarks, cannot be excluded, and leads to misinterpretation to a high percentage. The direction lesion healing can also affect its visibility on the periapical radiograph. In the case of lesion reduction in the buccolingual direction or within the cancellous bone, it does not have to be visible in a 2D radiograph. The size of the lesion can also be affected by the film or tube head orientation. However, CBCT is detecting 20–39% more posttreatment lesions than periapical radiographs. As a result, radiographic evaluation after the root canal treatment by the periapical radiographs can be false, which is why CBCT is recommended in those cases [15]. Different literatures state the success of healing outcomes after different time spans, attested by volumetric changes of the apical lesions [10,11,12,14,16,17,20,21]. Some literatures state that 12 months, in many cases, shows a high rate of complete or nearly-completed healing (reconstruction of radio opaqueness at the side of lesion) [20], whereas other state that 12 months is not sufficient enough to talk about a complete lesion regression [12] and others even prolonged their scientific work up to 2 [16] or 4 years [14] in order to confirm complete healing. Therefore, the discussion of recall intervals, as well as the minimum time span of healing to be able to talk about complete lesion healing, is controversial. Regardless of the time span of recall appointments, we can clearly depict that CBCT significantly enlarges the option of monitoring any changes in accordance to size.

The limitation of our study was that we only included three randomized publications (low risk of bias) [10,12,56] and that only one publication decided to prolong the follow-up to 4 years. Moreover, the heterogenicity of the included publications made conducting meta-analysis impossible. The quality assessment score was moderate in the vast majority of the publications. This result is similar to the one achieved in a previous systematic review concerning epidemiologic data [8].

## 5. Conclusions

This systematic review investigated the publications concerning the volume of periapical lesions with the use of CBCT and demonstrated the significant benefits of volumetric measurements in solving problems with endodontic origin: from diagnostics to the assessment of the dynamics of treatment of apical periodontal disease. Furthermore, superimposition of anatomical structures, as well as poor sensitivity, can be ruled out; moreover, healing outcomes after different treatment approaches can be monitored more accurately and definitively. Three-dimensional CBCTPAVI index helps in planning correct treatment approaches and allowing for surveillance prediction and regression postoperatively. Volumetric measurements help the dental clinician to exactly determine the defect expansion three-dimensionally. This leads to more accurate and successful periapical surgery planning, especially according to its location and thus its outcome. Additionally, observation time after the endodontic treatment is not unified for clinicians. This study underlines that the volumetric research can help in the establishment of relevant and constant follow-up time. The volumetric measurements of AP will certainly become more common in everyday dental practice. Nevertheless, more research with longer observation time and unified methodology is needed to evaluate the volumetric measurements with the use of CBCT.

## Figures and Tables

**Table 1:** Search strategy in databases.

Database (*n*)	Search Strategy
PubMED (*n* = 110)	(Cone-Beam Computed Tomography[Mesh] OR cone-beam OR CBCT) AND (Periapical Periodontitis[Mesh] OR periapical) AND volume
Embase (*n* = 46)	(‘tooth periapical disease’/exp OR ‘tooth periapical disease’) AND (‘cone beam computed tomography’/exp OR ‘cone beam computed tomography’) AND (‘volume’/exp OR ‘volume’)
EBSCO Dentistry and Oral Sciences Source (*n* = 40)	periapical lesion AND cone beam computed tomography AND volume
Cochrane (*n* = 3)	‘‘periapical periodontitis” in Title Abstract Keyword AND “cone beam computed tomography” in Title Abstract Keyword AND “volume” in Title Abstract Keyword-(Word variations have been searched)

**Table 2:** Summarized characteristics of included studies.

Authors, Year, Country	Materials					Treatment	Purpose
	Patricipants			Teeth	Target Condition		
	Amount	Sex	Age (Years)				
Borden et al. [15] 2013 The Netherlands	42	32 F, 10 M	21–78	50 (9 anterior, 6 premolars, 35 molars)	Preoperative periapical radiolucency on both PA and CBCT scans	Single visit, crown down technique, lateral compaction and later warm vertical compaction techniques	The aim of this study was to measure the changes in lesion size after root canal treatments with PA and CBCT imaging and to assess the outcome based on these parameters.
Liang et al. [10] 2013 China	105	-	18–76	105 (incisors, canines, premolars)	Periapical lesions with radiographic evidence of bone loss	Single visit, crown down technique and warm vertical compaction filling	The aim of this study was to compare the outcome of a root canal treatment with and without additional ultrasonic activation of the irrigant.
Metska et al. [11] 2013 The Netherlands	37	12 F, 10 M	20–70	45	Apical periodontitis after completed endodontic treatment	Reendodontic treatment and lateral or warm vertical condensation	Assess by CBCT scans the volumetric changes of periapical radiolucencies in endodontically treated teeth 1 year after orthograde retreatment.
Cardoso et al. [53] 2015 Brazil	24	-	22–45	24	Teeth with primary endodontic infection, absence of periodontal pockets deeper than 4 mm	-	This clinical study was conducted to correlate the levels of endotoxins and bacterial counts found in primary endodontic infection with the volume of periapical bone destruction determined by CBCT analysis.
Zhang et al. [16] 2015 China	130	-	-	162	Periapical lesions 12 months after RCT	Crown down technique and warm vertical condensation	Assesses second-year volumetric changes in post-treatment periapical radiolucencies detected 1 year after treatment.
Aoki et al. [54] 2015 Brazil	43	29 F, 14 M	average age 54.6 ± 8.5	-	Periapical lesion after endodontic diseases	-	To test the reliability and reproducibility of 2 methods: manual and automated segmentation (using a threshold-based region growing algorithm) for measuring the volume of periapical lesions.
Rizzi-Maia et al. [12] 2016 Brazil	13	9 F, 4 M	18–58	26 (21 incisors, 1 canine, 4 premolars)	Teeth with pulp necrosis and visible periapical lesion	Single session: lateral condensationTwo sessions: Ca(OH)_2_ and then filled with lateral condensation	To compare root canal treatment of teeth with AP in a single or two visits, using CBCT.
Kamburoglu et al. [20] 2017Turkey	21	14 F, 7 M	18–52	21 (molars)	Periapical lesions with mucosal thickening more or equally 2 mm	Two sessions with Ca(OH)_2_ dressing and single cone gutta percha technique	To obtain linear and volumetric measurements of lesion dimensions in maxillary first molars with periapical pathology and to measure maxillary sinus mucosal thickening in the vicinity of periapical lesions before and 1 year after endodontic treatment by using CBCT.
Filho et al. [13] 2018 Brazil	21	-	average age 36.67 ± 11.21	35 (30 incisors, 2 canines, 3 premolars)	Pulp necrosis and radiographically visible chronic apical periodontitis	-	The study aimed to correlate the Periapical Index, obtained by way of periapical radiographs, with the volume of chronic periapical lesion, obtained through CBCT, in the permanent teeth.
Jalali et al. [17] 2019 USA	48	22 F, 26 M	older than 18	48	Acute or chronic apical abscesses	-	Evaluate the size and pattern of bone loss in patients with acute apical abscess and chronic apical abscess using CBCT images.
Sönmez et al. [21] 2019 Turkey	33	23 F, 10 M	18–62	33 (19 incisors, 1 canine, 10 premolars, 3 molars)	Periapical lesions which had been determined as granulomas or periapical cysts	Two sessions with Ca(OH)_2_ dressing and single cone gutta percha technique	To evaluate and compare the diagnostic potential of high-resolution ultrasound with periapical radiographs (PR) and CBCT in assessing granulomas and radicular cysts.
Garcia-Font et al. [3] 2020 UK	-	-	18–79	131 (16 premolars, 115 molars)	Periapical lesions of primary and secondary endodontic lesions	Primary and secondary endodontic infections	CBCT was used to evaluate the differences in the mucosal thickness of the Schneiderian membrane in primary and secondary endodontic lesions.
Zhang et al. [14] 2020 China	80	68 F, 29 M	≤45 and >45	97 (46 anterior, 22 premolars, 29 molars)	Teeth, which had to undergo nonsurgical root treatment	Reendodontic treatment, crown down technique, single visit and vertical warm gutta percha technique	To investigate the 4-year outcome and prognostic factors of nonsurgical root canal retreatment determined by measuring the volumetric change of periapical radiolucencies on CBCT scans.
Boubaris et al. [18] 2021 Australia	91	52 F, 39 M	average age 55 ± 5.15	273 roots (44 incisors, 19 canines, 62 premolars, 148 molars)	Periapical lesion after endodontic diseases	-	To evaluate the variations in the volume of periapical lesions scored using a cone-beam computed tomographic periapical index (CBCTPAI) and to develop a new volume-based periapical index.
Boubaris et al. [19] 2022 Australia	113	66 F, 47 M	mean age 56	261 roots (53 incisors, 15 canines, 48 premolars, 145 molars	Teeth with periapical radiolucency.	-	The aim of this study was to assess the sphericity of periapical radiolucent lesions and its relation to the CBCTPAVI.
Machut et al. [55] 2022 Poland	36 (3 patients into both groups)	10 F, 9 M (study group); 9 F, 10 M (control group)	average age Study group: 33.7 Control group: 30.0	40 Study group (11 incisors, 3 canines, 4 premolars, 2 molars). Control group (9 incisors, 2 canines, 6 premolars, 3 molars)	Apical periodontitis	Modified crown-down technique. Application of A-PRF below thecemento-dentinal junction. Final obturation by the thermoplastic method with calibrated gutta-percha cone and AH-plus sealer.	The aim of this study is to compare six-month follow-ups of periapical lesion healing after one-visit RCT with A-PRF+ application vs. two-visit RCT with inter-appointment calcium hydroxide dressings.
Toia et al. [56] 2022 Brazil	40	12 F, 28 M	12–60	40 (29 incisors, 2 canines, 9 premolars)	Posttreatment apical periodontitis	One or two visit root canal treatments. Crown down technique in both groups. Two visit treatment: Ca(OH)_2_ for 14 days. Single cone technique in both groups.	The aim of this study was to compare the endodontic retreatment of root-filled teeth with PTAP performed in 1 visit vs. 2 visits on the reduction of microbial load.

F—females, M—males.

**Table 3:** The most important parameters of the CBCT apparatus are from included studies.

	Analysis CBCT								
Authors	Apparatus	Apparatus Parameters							Programme
		Voltage (kVp)	Current (mA)	Field of View (cm)	Voxel Size (mm^3^)	Slice Dimensions (pixels)	Thickness of the Cut Layer (mm)	Segmentation Method	
Borden et al. [15] 2013	Gendex CB-500; KaVo Dental GmbH, Biberach, Germany	120	5	-	-	-	0.125–0.2	Semiautomatic	Amira Software v.5.4.3
Liang et al. [10] 2013	3DX-Accuitomo CBCT scanner (J. Morita Mfg Corp, Kyoto, Japan)	80	4 to 5	4 × 4	-	-	-	Semiautomatic	Amira Software v.5.4.3
Metska et al. [11] 2013	Pre op: NewTom 3G (QR SLR, Verona, Italy) Post op: NewTom 5G	1. Pre op: 110 2. Post op:110	1.Pre op: 3.90–5.6 2. Post op: 3.76–6.43	1. Pre op: 9 inch 2. Post op: 8 × 8	-	-	-	Semiautomatic	AMIRA software v.5.3.4
Cardoso et al. [53] 2015	I-CAT Next Generation (Imaging Science International, Hatfield, PA, USA)	-	-	8 × 8	0.2	-	0.2	Automatic	NEMOTEC software
Zhang et al. [16] 2015	3DX-Accuitomo scanner (JMorita Mfg Corp, Kyoto, Japan)	80	4–5	4 × 4	-	-	-	Manual	Amira software v. 5.4.3
Aoki et al. [54] 2015	I-CAT Classic (Image Sciences International, Hatfield, PA, USA)	120	8	16 × 6	0.25	-	-	Manual: 120 s Automatic: 50 s	OsiriX ImplantViewer 3.006
Rizzi-Maia et al. [12] 2016	I-CAT Next Generation device (Imaging Sciences International, Hatfield, PA, USA)	-	-	8 × 8	0.2	-	0.2	Automatic	Amira software v.5.3.3
Kamburoglu et al. [20] 2017	Kodak CS 9300 3D (Carestream Health Co, Rochester, NY, USA)	80	8	-	0.09	-	-	Manual	3D Doctor
Filho et al. [13] 2018	Next Generation I-CAT (Imaging Sciences International, Hatfield, PA, USA)	-	-	8 × 8	0.2	-	0.2	Automatic	Amira software v.5.3.3
Jalali et al. [17] 2019	CS 9300 device (Carestream Health, Rochester, NY, USA)	60–90 kV	2–15	-	0.09	-	-	Automatic	Mimics Innovation Suite Version 19 software
Sönmez et al. [21] 2019	Planmeca Promax 3D max CBCT unit (Planmeca, Helsinki, Finland)	90	7	55 × 50 mm	0.1	-	-	Manual	3D- Doctor
Garcia-Font et al. [3] 2020	ProMax 3Ds (Planmeca OY, Helsinki, Finland)	84 kV	8.0	19–24 micro SV	0.2	-	0.2	-	Planmeca Romexis Viewer
Zhang et al. [14] 2020	-	-	-	-	-	-	-	Manual	Amira software v. 5.4.3
Boubaris et al. [18] 2021	-	-	-	-	-	-	-	Semiautomatic	Mimics Research v.21.0.0.406
Boubaris et al. [19] 2022	Carestream CS9600 CBCT Scanner; Carestream Dental LLC, Atlanta, GA, USA)	-	-	-	-	-	-	Semiautomatic	Mimics Research v.21.0.0.406
Machut et al. [55] 2022	CS 3D Imaging v3.5.18 Software (Carestream Health Inc., Trophy, Croissy-Beaubourg, France)	-	-	-	-	-	-	-	ITK-SNAP
Toia et al. [56] 2022 Brazil	I-cat CBCT (Next Generation; Imaging Science International, Hatfield, PA, USA)	120	36.15	16 × 13	0.25	-	-	Semiautomatic	ITK-SNAP v. 3.8.0 software (Cognitica, Philadelphia, PA, USA)

**Table 4:** Summarized results of included studies.

Authors			Preoperative Volume (mm^3^)		Time of Postoperative Volume Measurement		Postoperative Volume (mm^3^)		Conclusion	
Borden et al. [15] 2013			1.0–281.5		10–37 months		-		Lesion changes after root canal treatments determined with 3D volumetric CBCT data and two-dimensional PA data were different, and, thus, the outcome determined with PA could be untrue.	
Liang et al. [10] 2013			1.5–375.4		10–19 months		0.00–176.20		Root canal treatments with and without additional ultrasonic activation of the irrigant equally contributed to periapical healing and resulted in a high percentage of absence and reduced lesions, which is seen on outcomes of volumetric measurements.	
			Median volume of the lesion in the ultrasonic group: 26.6				-			
			Median volume of the lesion in the syringe group: 31.8				-			
Metska et al. [11] 2013			2.26–998.58		1 year		0.00–1215.14		The volumetric measurements revealed a reduction of the size of periapical radiolucencies in more than half of the teeth 1 year after orthograde retreatment.	
Aoki et al. [54] 2015			Lack of precise data		-		-		Automated segmentation with a region growing algorithm is faster and slightly more reliable to calculate the volume of periapical lesions.	
Cardoso et al. [53] 2015			100		-		-		Findings revealed that the levels of endotoxins found in root canal infection are related to the volume of periapical bone destruction determined by CBCT analysis.	
Zhang et al. [16] 2015			2.6–339.7		1 year		0.8–174.6		The volumes of post-treatment periapical radiolucencies detected 1 year after treatment in 63% of these teeth showed significant decreases in size during the second year, including complete resolution of the radiolucency in 13 teeth (22%). Thus, the healing of apical periodontitis is a dynamic process that takes time.	
					2 years		0.00–248.0			
Rizzi-Maia et al. [12] 2016			One session treatment 73.47 (11.119–182.48)		1 year		27.73 (1.07–101.16)		Cone beam-computed tomography imaging made 12 months posttreatment did not show complete repair in any of the teeth, based on the volumetric assessment of the lesion, suggesting that this follow-up period is not sufficient for the occurrence of complete lesion regression.	
			Two session treatment 65.94 (8.27–238.01)				12.84 (0.11–29.53)			
Kamburoglu et al. [20] 2017			74.95		1 year		19.38		Within the limitations of this study, we found a significant reduction in periapical lesion width, lesion height, surface area, and volume in maxillary molar teeth, along with adjacent sinus mucosal thickening by using CBCT and specific software 1 year after endodontic treatment.	
			Mucosal thickening: 5.7 mm				2 mm			
Jalali et al. [17] 2019			AAA: 109		-		-		Cortical fenestration is fundamental for the development of CAA. However, periradicular lesions without evident cortical fenestration can still cause AAA and fascial space involvement.	
			CCA: 233							
Sönmez et al. [21] 2019			Using ultrasound 394.85		6 months		112.22		Although lesion depth, surface area, and volume are underestimated, in which CBCT is more accurate, lesion width and pathology as well as treatment outcomes are accurately assessed using ultrasound.	
			Using CBCT 736.32				not measured			
Garcia-Font et al. [3] 2020			Primary infection: lesion volume: 0.05 cm^3^, mucosal thickness: coronal view 5.41		-		-		Secondary endodontic infections showed a more increased volume than that in primary endodontic infections. Furthermore, a significant association was noted between the volume and membrane thickness, revealing a greater volume increase in the thickness of the membrane in the primary and secondary infections in 2 planes.	
			Secondary infection: lesion volume 0.12 cm^3^; mucosal thickness: coronal view 3.4		-		-			
Zhang et al. [14] 2020			0.7–451.5		4 years		0.00–30.6		The 4-year outcome of endodontic retreatment was predictable, with a significant volumetric reduction in periapical radiolucencies.	
Machut et al. [55] 2022			-		6 months		-		The results of 3D radiographic healing assessments of RCT using modified criteria were different from those based on CBCT-PAI criteria. In the 6-month follow-up, CBCT scans showed a better healing tendency in patients in the study group than in the control group. The volumes of apical radiolucency were, on average, reduced by 85.93% in the study group and by 72.31% in the control group.	
Toia et al. [56] 2022			One visit treatment: 54 (10–375) Two visits treatment: 60 (30–470)		18 months		One visit treatment: 10 (0–30) Two visits treatment: 5.5 (1–48)		In conclusion, 18 months after endodontic retreatment of root-filled teeth with PTAP, no significant differences were observed in the reduction of periapical lesion volume between teeth treated in 1 visit and those treated in 2 visits using Ca(OH)_2_ for 14 days. Even with the remaining content of LPS and LTA bacteria, it was possible to observe a significant reduction in the volume of periapical lesions in both groups after 18 months of treatment.	
	Volume (mm^3^)								Total	
	PAI 0	PAI 1		PAI 2	PAI 3	PAI 4	PAI 5	PAI 6		
Filho et al. [13] 2018	-	-		36.42	55.83	76.1	143.78	-	70.72	Radiographic evaluation of periapical lesions must be carried out with caution, as it may not reflect the lesion’s volumetric characteristics.
	CBCTPAVI 0	CBCTPAVI 1		CBCTPAVI 2	CBCTPAVI 3	CBCTPAVI 4	CBCTPAVI 5	CBCTPAVI 6		
Boubaris et al. [18] 2021	0	0.01–0.2		0.21–0.7	0.71–8.00	8.01–70.00	70.01–100.00	100.01+	-	The method described in this article is a valid option for scientific inquiry, thus the continuing development of existing imaging software will allow for automation of the extraction of volume data from cone-beam images.
				Sphericity						
	CBCTPAVI 0	CBCTPAVI 1		CBCTPAVI 2	CBCTPAVI 3	CBCTPAVI 4	CBCTPAVI 5	CBCTPAVI 6		
Boubaris et al. [19] 2022	85.7%	85.7%		89.1%	80.4%	77.8%	77.8%	59.6%		Periapical lesions of endodontic origin are mostly semi-spherical (51–78%) in their spread, and as CBCTPAVI score increases, sphericity decreases, indicating that larger lesions expand less uniformly compared with smaller lesions. Clinicians should be aware that lesions of increased volume have less sphericity, and are thus elongated or stretched in 1 or more anatomic plane.

AAA—acute apical abscesses; CCA—chronic apical abscesses; CBCTPAVI—cone-beam computed tomographic periapical volume index.

**Table 5:** Risk of bias.

Borden et al. (2013) Netherlands [15]	Liang et al. (2013) China [10]	Metska et al. (2013) Netherlands [11]	Cardoso et al. (2015) Brazil [53]	Zhang et al. (2015) China [16]	Aoki et al. (2015) Brazil [54]	Rizzi-Maia et al. (2016) Brazil [12]	Kamburoglu et al. (2017) Turkey [20]	Filho et al. (2018) Brazil [13]	Jalali et al. (2019) USA [17]	Sönmez et al. (2019) Turkey [21]	Garcia-Font et al. (2020) United Kingdom [3]	Zhang et al. (2020) China [14]	Boubaris et al. (2021) Australia [18]	Boubaris et al. (2022) Australia [19]	Machut et al. (2022) Poland [55]	Toia et al. (2022) Brazil [56]	
-	+	-	-	-	-	+	-	-	-	-	-	-	-	-	-	+	Random or pseudorandom sample
+	+	+	+	+	+	+	+	+	+	+	+	+	+	+	+	+	Clear inclusion criteria
-	-	-	-	-	-	-	-	-	-	-	-	-	-	-	-	-	Confounding factors
+	+	+	+	+	+	+	+	+	+	+	+	+	+	+	+	+	Objective criteria
+	+	+	+	+	+	+	+	+	+	+	+	+	+	+	+	+	Description of comparisons
+	+	+	-	+	-	+	-	-	-	-	-	+	-	-	+	+	Follow up in sufficient time
-	-	-	-	-	-	-	-	-	-	-	-	-	-	-	-	-	Withdrewed patients
+	+	+	+	+	+	+	+	+	+	+	+	+	+	+	+	+	Outcomes measurments
+	+	+	+	+	+	+	+	+	+	+	+	+	+	+	+	+	Statistical analysis
6/9	7/9	6/9	5/9	6/9	5/9	7/9	5/9	5/9	5/9	5/9	5/9	6/9	5/9	5/9	6/9	7/9	YES
M	L	M	M	M	M	L	M	M	M	M	M	M	M	M	M	L	Risk of bias

“+”—yes; “-”—no; L—low; M—moderate.

## Data Availability

Not applicable.
